# S1P Lyase Deficiency in the Brain Promotes Astrogliosis and NLRP3 Inflammasome Activation via Purinergic Signaling

**DOI:** 10.3390/cells12141844

**Published:** 2023-07-13

**Authors:** Shah Alam, Sumaiya Yasmeen Afsar, Maya Anik Wolter, Luisa Michelle Volk, Daniel Nicolae Mitroi, Dagmar Meyer zu Heringdorf, Gerhild van Echten-Deckert

**Affiliations:** 1 LIMES Institute for Membrane Biology and Lipid Biochemistry, Kekulé-Institute, University of Bonn, 53115 Bonn, Germany; shah.bonn@outlook.com (S.A.); sumaiya.shah@outlook.com (S.Y.A.); maya.a.wo@gmail.com (M.A.W.); daniel.mitroi@abbvie.com (D.N.M.); 2 Institute for General Pharmacology and Toxicology, University Hospital, Goethe University Frankfurt am Main, 60590 Frankfurt am Main, Germany; volk@med.uni-frankfurt.de (L.M.V.); heringdorf@med.uni-frankfurt.de (D.M.z.H.)

**Keywords:** sphingosine 1-phosphate (S1P), S1P-lyase (SGPL1), astrogliosis, P2Y1 receptor (P2Y1R), neuroinflammation, NLRP3 inflammasome, DDX3X, calcium, calbindin

## Abstract

Astrocytes are critical players in brain health and disease. Brain pathologies and lesions are usually accompanied by astroglial alterations known as reactive astrogliosis. Sphingosine 1-phosphate lyase (SGPL1) catalysis, the final step in sphingolipid catabolism, irreversibly cleaves its substrate sphingosine 1-phosphate (S1P). We have shown that neural ablation of SGPL1 causes accumulation of S1P and hence neuronal damage, cognitive deficits, as well as microglial activation. Moreover, the S1P/S1P-receptor signaling axis enhances ATP production in SGPL1-deficient astrocytes. Using immunohistochemical methods as well as RNA Seq and CUT&Tag we show how S1P signaling causes activation of the astrocytic purinoreceptor P2Y1 (P2Y1R). With specific pharmacological agonists and antagonists, we uncover the P2Y1R as the key player in S1P-induced astrogliosis, and DDX3X mediated the activation of the NLRP3 inflammasome, including caspase-1 and henceforward generation of interleukin-1ß (IL-1ß) and of other proinflammatory cytokines. Our results provide a novel route connecting S1P metabolism and signaling with astrogliosis and the activation of the NLRP3 inflammasome, a central player in neuroinflammation, known to be crucial for the pathogenesis of numerous brain illnesses. Thus, our study opens the door for new therapeutic strategies surrounding S1P metabolism and signaling in the brain.

## 1. Introduction

Sphingosine 1-phosphate (S1P) is an evolutionarily conserved catabolic intermediate of sphingolipid metabolism that exerts multiple cellular functions either as a ligand of a subfamily of five G-protein-coupled receptors (S1PR _1–5_) or acting intracellularly as a second messenger [1]. There is convincing experimental evidence for the crucial role of S1P in the regulation of diverse fundamental processes in the brain, including neural development, differentiation, migration, and survival [2,3]. The amount of S1P in the brain far exceeds that found in any other organ, such as the liver or spleen [4]. Presumably S1P is enriched in the brain simply because this organ contains the highest concentration of complex sphingolipids in the body [5], which, being consequently metabolized, produce considerable amounts of this bioactive catabolite. S1P is the degradation product of ceramide, the membrane anchor of all sphingolipids. Deacylation of ceramide yields sphingosine that is phosphorylated by sphingosine kinases (SKs) to form S1P. From the two isoforms of SKs described so far, SK2 is the predominant one in the brain [6]. The interplay of SKs with S1P phosphatases (SPP1 and SPP2) enables a fine-tuned regulation of the S1P concentration in different brain regions. Most important for the amount of S1P in the tissue, however, is S1P-lyase (SGPL1), which irreversibly cleaves S1P to ethanolamine phosphate and hexadecenal [7]. In humans, SGPL1 is encoded by *SGPL1*, which is prone to mutations found in several cancers [8] and also in a variety of pathologies that include peripheral and central neurological defects, collectively referred to as S1P-lyase insufficiency syndrome (SPLIS) [9], which can lead to early infant death [10].

Despite the established role of S1P in brain development [11,12], its involvement in neurodegenerative processes remains a topic of ongoing discussion [2,13]. Conflicting findings have emerged regarding the potential neuroprotective [14,15,16] or neurotoxic effects of S1P in the brain [17,18,19]. To gain insights into the function of S1P in the brain, we generated a mouse model with specific inactivation of SGPL1 (sphingosine-1-phosphate lyase) in neural cells (SGPL1^fl/fl/Nes^), resulting in substantial S1P accumulation in the brain [20]. We have previously observed significant alterations in synaptic architecture and plasticity in the SGPL1^fl/fl/Nes^ mouse model [20]. Additionally, we also found that the early stages of neuronal autophagy were impaired, leading to the accumulation of neurodegenerative biomarkers [21]. These changes were accompanied by behavioral deficits, including cognitive impairments and motor coordination issues in SGPL1^fl/fl/Nes^ mice [20,21]. Moreover, the neuronal accumulation of S1P led to increased cytosolic calcium levels and hyperphosphorylation of tau at sites relevant to disease progression [22]. Furthermore, the activation of microglial cells, propagating neuroinflammation and neuropathology, was triggered in response to neuronal damage in the SGPL1-deficient brains [23]. Notably, astrocyte-derived S1P emerged as a critical factor in the activation of microglia in neural-specific SGPL1-deficient brains [23]. Collectively, these findings shed light on the intricate role of S1P in the brain and provide evidence for its impact on synaptic function, neuronal autophagy, neurodegenerative processes, and the interplay between different cell types in the context of SGPL1 deficiency. Furthermore, the S1P/S1PR_2/4_ signaling axis altered glucose metabolism, thus increasing astrocytic ATP [24]. We now investigated the consequences of these molecular and physiological changes induced by S1P in SGPL1-deficient brains and in primary cultured astrocytes. Astrocytes surpass neurons by more than five-fold in number [25]. Their significance in governing early neurodevelopmental processes has been well-established [26]. Furthermore, their indispensable contribution to maintaining normal neural activity in a healthy brain and their rapid responsiveness to various forms of brain injury are increasingly acknowledged [25]. The response of astrocytes to brain insults is generally referred to as reactive astrogliosis [25]. Up-regulation of the expression of glial fibrillary acidic protein (GFAP) is one hallmark of moderate reactive gliosis [26]. In the present study, we therefore monitored the expression of GFAP in the brain of SGPL1^fl/fl/Nes^ mice as a first indication for reactive astrogliosis [27]. As SGPL1-deficient astrocytes were recently reported to generated increased ATP amounts [24], we explored the potential role of a purinergic mechanism for astrogliosis and hence for calcium homeostasis and neuroinflammation known to be intimately associated with this process.

## 2. Materials and Methods

### 2.1. List of Abbreviations

CNS: central nervous system; S1P: sphingosine 1-phosphate; SGPL1: S1P-lyase; S1PR: S1P receptor; GFAP: glial fibrillary acidic protein; P2Y1R: P2 purinoceptor subtype Y1; NLRP3: nucleotide-binding domain leucine-rich-containing family pyrin domain-containing-3; DDX3X: DEAD-box helicase 3 X-linked; IL-1β: interleukin-1-beta; TNFα: Tumor Necrosis Factor α.

### 2.2. Antibodies and Chemicals

Monoclonal antibodies against GFAP (3670), NLRP3 (15101), Caspase1 (3866), IL-1β (12242), Ddx3 (2635), Calbindin (13176), and β-actin (4967) were purchased from Cell Signaling Technology (Danvers, MA, USA). Polyclonal antibody against P2Y1R was from Thermo Fisher Scientific, Waltham, MA, USA, BS-1204R. Secondary antibodies, HRP-linked anti-rabbit and anti-mouse IgG were purchased from Cell Signaling Technology (7074 and 7076, Danvers, MA, USA). MRS2179 and MRS2905 were obtained from Tocris (0900, 5633, Wiesbaden-Norderstedt) and JTE013 and CYM55380 from Sigma-Aldrich (J4080 and SML1066, respectively, St. Louis, MO, USA). CYM5520 and CYM50308 were from Cayman Chemical Company (17638 and 14667, respectively, Ann Arbor, MI, USA).

### 2.3. Animals

The SGPL1^fl/fl/Nes^ mouse model was created as described previously [20]. To generate this model, mice with both Sgpl1 alleles containing “floxed” exons 10–12 (referred to as SGPL1^fl/fl^) were bred with Nes-Cre1 transgenic mice, where the nestin promoter regulated the expression of Cre recombinase. Through this breeding process, SGPL1^fl/fl/Nes^ mice were obtained, in which the Cre recombinase excised the “floxed” exons, resulting in the removal of SGPL1 specifically in neural cells. To serve as a comparison in all experiments, SGPL1^fl/fl^ mice were used as control subjects. The mice were raised under standard conditions at the LIMES Institute of Bonn University. Complying with the 3R ethical principle (Replace, Reduce, Refine), we reduced the number of animals whenever possible and replaced them by using cultured astrocytes. Furthermore, we used strategies that complemented each other, e.g., analysis of the effects of both agonists and antagonists.

### 2.4. Cell Culture

To obtain primary astrocyte cultures, mixed glial cultures were prepared from the cortices of postnatal pups ranging from P1 to P4. The procedure involved decapitating the pups and removing both the skull and skin along the midline following careful extraction of brain in ice-cold HBSS buffer. The cerebellum and meninges were then removed using forceps and transferred to a separate 15 mL tube filled with 1–2 mL of HBSS (without Ca^2+^ and Mg^2+^) and kept on ice until all brains were dissected. Following the dissection of both control and SGPL1-deficient brains, HBSS buffer was aspirated, and 1–2 mL of 0.05% trypsin-EDTA was added. The tubes were incubated in a water bath at 37 °C for 10 min with constant shaking. To neutralize the effect of trypsin-EDTA, 1–2 mL of prewarmed cell culture medium was added, and the cortices were mechanically dissociated by pipetting up and down using a sterile 10 mL pipette. The tubes were then centrifuged for a brief duration, and the supernatant was carefully aspirated and replaced with 1–2 mL of prewarmed cell culture medium (DMEM). The cell suspension was finally transferred to a T25 cell culture flask containing 5 mL of prewarmed cell culture medium to replenish the cells with essential nutrients and growth factors. The cell culture flask housing the cells was incubated overnight in a humidified cell culture incubator, maintaining a temperature of 37 °C enriched with 5% CO_2_. The following day, the culture medium was carefully removed, and the cells were subjected to a thorough washing with prewarmed sterile PBS in order to eliminate any remnants of cell debris. Following this cleansing process, the cells were further incubated in a fresh 5 mL of complete culture medium, which was refreshed every 2–3 days to provide optimal conditions for cell growth and vitality. After about 10 days, a dense layer of astrocytes formed, accompanied by the presence of loosely growing microglia and oligodendrocytes atop this astrocytic layer. Thereafter, approximately 25 days into the culture period, the astrocytes reached a stage suitable for further experimental application. Prior to conducting experiments, a thorough separation of microglia and oligodendrocyte precursor cells (OPCs) was achieved through vigorous shaking, ensuring the isolation of astrocytes for further study. The medium was then removed, and astrocytes were used for experiments after 24 h as needed. Note that each T25 flask contained cortical astrocytes from one pup (independent litter). For all experiments, astrocytes from at least 3 different litters corresponding to 3 different T25 flasks were used.

### 2.5. Western Immunoblotting

Tissue and cell samples were crushed in RIPA buffer (Thermo Fisher Scientific, 89900) and incubated for 1 h on ice with occasional vigorous vortexing. They were then centrifuged at 14,000 rpm at 4 °C for 45 min. Cell pellets were thawed on ice and mixed with RIPA lysis buffer (150 µL) using a pipette and then incubated for 1 h on ice with vigorous mixing. They were then centrifuged at 13,000 rpm for 45 min at 4 °C. Protein concentration in the supernatants was determined using Nanodrop (Thermo Fisher Scientific, ND-2000). Lysates were mixed with Laemmli buffer in a 1:4 ratio (Bio-RAD Laboratories, Munich, Germany, 1610747) and heated at 95 °C for 5 min before being loaded onto SDS-PAGE gel. Proteins were separated by SDS-PAGE in running buffer (25 mM Tris, pH 8.3, 192 mM glycine, 0.1% SDS) and transferred onto nitrocellulose membranes (Porablot NCL; Macherey-Nagel, Thermo Fisher Scientific, 741290) in transfer buffer (50 mM Tris, pH 9.2, 40 mM glycine, 20% methanol) at 4 °C and 400 mA for 2 h. The membranes were then blocked with Blocker BSA (Thermo Fisher Scientific, 37520) in TBS-Tween 20 and incubated overnight with the primary antibody at 4 °C. After washing, the membranes were incubated for 1 h with an HRP-conjugated secondary antibody and detected with Western BLoT Chemiluminescence HRP Substrate (TAKARA Bio, Saint-Germain-en-Laye, France, T7101B) using the VersaDoc 5000 imaging system (Bio-Rad, Hercules, CA, USA). β-actin was used as the loading control, and, if a protein of similar molecular weight to β-actin was present on the blot, the blots were stripped before being used for β-actin imaging. Quantification and statistical analysis were performed using ImageJ 1.51j8 and GraphPad Prism 9 programs.

### 2.6. RNA Isolation and Real-Time PCR

Up to 1 µg of total RNA (isolated with EXTRAzol from Blirt, Gdańsk, Poland, EM30-200) was used for reverse transcription with the ProtoScript^®^ II First Strand cDNA Synthesis kit (New England Biolabs, E6560L, easy protocol). The resulting total cDNA was then applied to real-time PCR (CFX96real time PCR, Bio-Rad). The primers for real-time PCR were obtained from Invitrogen (Carlsbad, CA, USA) and designed using the online tool from NCBI BLAST. They were listed as follows: name: forward primer (for) and reverse primer (rev): β-actin: 5′-CTTTGCAGCTCCTTCGTTGC-3′ (for) and 5′-CCTTCTGACCCATTCCCACC-3′ (rev); TNF: 5′-TTGACCTCAGCGCTGAGTTG-3′ (for) and 5′-CCTGTAGCCCACGTCGTAGC-3′ (rev); IL-6: 5′-GGAAATCGTGGAAATGAG-3′ (for) and 5′-GCTTAGGCATAACGCACT-3′ (rev); IL-11: 5′-ATGAACTGTGTTTGTCGCCTG-3′ (for) and 5′-CAGCTAGTTGCCGTGTGTCT-3′ (rev); IL-15: 5′-CTCTGCGCCCAAAAGACTTG-3′ (for) and 5′-GGTGGATTCTTTCCTGACCTC-3′ (rev); IL-18: 5′-GTTTACAAGCATCCAGGCACAG-3′ (for) and 5′-GAAGGTTTGAGGCGGCTTTC-3′ (rev). The reactions were performed at 95 °C for 30 s, 95 °C for 10 s, and 60 °C for 1 min. Relative normalized mRNA expression was obtained from real-time qPCR. The fold increase or decrease was determined relative to controls after normalizing to β-actin as a housekeeping gene, and statistical analysis was performed via GraphPad Prism 9 program.

### 2.7. Immunocytochemistry

Following approximately 21 days of growth in T25 flasks, the cultured cells were transferred onto coverslips and allowed to proliferate for an additional 8–10 days. Subsequently, the coverslips containing the astrocytes were gently rinsed three times with PBS at room temperature. To ensure fixation, the cells were then exposed to chilled methanol (−20 °C) for a duration of 5 min. Between each subsequent step, the cells were consistently rinsed three times with PBS. Next, to prevent non-specific binding, the cells were subjected to a blocking process with 20% (*v*/*v*) normal goat serum in PBS for a duration of 30 min, incubated overnight at 4 °C with primary antibodies, and diluted to a concentration of 1:200 in PBS. After the primary antibody incubation, the cells underwent a 50 min incubation at room temperature with anti-rabbit/mouse Alexa Fluor 488 (1:300)-conjugated secondary antibodies. Finally, to enable visualization and microscopic analysis, the cells were finally embedded in Fluoromount G medium, supplemented with DAPI.

### 2.8. Immunohistochemistry

The isolated brain samples were promptly snap frozen in liquid nitrogen to preserve their molecular integrities. Cryo-sectioning technique was employed to produce 10 µm sagittal sections, which were then carefully mounted on Superfrost Plus positively charged microscope slides. To ensure fixation, the brain sections were exposed to ice-cold 4% (*v*/*v*) paraformaldehyde in PBS for a duration of 5 min. Subsequently, permeabilization of the tissue sections was achieved by treating them with 0.1% (*v*/*v*) Triton X-100 in PBS for 30 min at room temperature. Following the permeabilization step, the tissue sections underwent a blocking procedure using 20% (*v*/*v*) normal goat serum in PBS for 30 min to prevent non-specific binding. The sections were then incubated overnight at 4 °C with primary antibodies and diluted at a ratio of 1:200 in PBS containing 0.5% lambda-carrageenan and 0.02% sodium azide. After thorough washing to remove any unbound primary antibodies, the brain sections were next incubated with a Cy3-conjugated anti-rabbit secondary antibody, which was diluted at a ratio of 1:300 in PBS with the same additives as mentioned above, for 1 hr at room temperature. Finally, the antibody-labeled brain sections were embedded in Fluoromount G medium containing DAPI for subsequent microscopic analysis. The microscopic examination was performed using a Keyence compact fluorescence microscope from the Keyence microscope BZ-X series, enabling visualization and analysis of the labeled brain sections. 

### 2.9. ADP and ATP Measurement

The ADP level was determined using the Sigma-Aldrich kit (MAK135). Briefly, the ADP immediately reacts with the substrate D-luciferin in the presence of luciferase to produce light. The light intensity represents the extracellular ADP concentration directly.

The concentration of ATP in the extracellular medium was determined using the Sigma-Aldrich kit (MAK190, Darmstadt, Germany). Briefly, the light produced by the reaction of ATP with the substrate D-luciferin in the presence of luciferase denotes the ATP concentration.

### 2.10. Enzyme-Linked Immunosorbent Assay (ELISA)

The concentrations of IL-6 and TNFα in primary cultured astrocytes and their culture media were measured using ELISA kits from Invitrogen™ eBioscience™, following the manufacturer’s guidelines. The Mouse IL-6 ELISA Ready-SET-Go™ Kit (15511037) was used to measure IL-6, and the Mouse TNFα ELISA Ready-SET-Go™ Kit (88-7324-86) was used to measure TNFα. The results are expressed as pg/mL and pmol/mg, respectively.

### 2.11. Measurement of Intracellular Calcium Concentration ([Ca^2+^]_i_)

Basal [Ca^2+^]_i_ was measured with fura-2, as described in [28]. Astrocytes seeded onto 8-well chambered cover slides (μ-slide; ibidi GmbH, Martinsried, Germany). The cells were loaded with 4 μM fura-2/AM (Molecular Probes/Invitrogen #F1221) in Hank’s balanced salt solution (HBSS; 118 mM NaCl, 5 mM KCl, 1 mM CaCl_2_, 1 mM MgCl_2_, 5 mM D-glucose and 15 mM HEPES pH 7.4) for ~45 min at room temperature. [Ca^2+^]_i_ was measured with a Till Photonics Calcium Imaging system equipped with a rapid filter-switching microscope light source (Oligochrome), an EMCCD camera (Andor Luca R), filters for excitation of fura-2 (340/26 and 387/11) and GFP (470/22), emission filters (beam splitter 495, bandpass 525/45), and the Live Acquisition software version 2.2 (Till Photonics GmbH, Gräfelfing, Germany). The system was coupled to a Zeiss Axiovert 135 TV inverted microscope. Mean fluorescence of single cells was monitored in defined regions of interest, and data were evaluated with the Live Acquisition software. Graphical presentations and statistical analyses were performed with GraphPad Prism 9 software.

Cellular responses to agonists were measured by calcium imaging, essentially as described in [29] with small alterations. Astrocytes grown on 8-well chambered cover slides were loaded with 4 µM fluo-4/AM (Molecular Probes/Invitrogen, F14201) in HBSS for ~30 min at room temperature. Thereafter, they were washed with HBSS and analyzed by confocal laser scanning microscopy, using a Zeiss LSM510 Meta system equipped with a Zeiss Axiovert 200 microscope and a Plan-Neofluar 40X/1.3 oil immersion objective (Carl Zeiss MicroImaging GmbH, Göttingen, Germany). The 488 nm laser line was used for excitation, whereas emission was recorded with a 505 nm long pass filter. Time series were generated with 1 image/s, and the mean fluorescence of the single cells was monitored after defining appropriate regions of interest. Microscopic images were analyzed with the ZEN 2009 software (Carl Zeiss MicroImaging GmbH, Göttingen, Germany). Graphical presentations and statistical analyses were performed with Prism 9. For quantification of [Ca^2+^]_i_ measurements, the fluorescence at a given time point was normalized to baseline fluorescence, defined as mean fluorescence during the first ~15 s (F/F_0_). Maximal increases in fluorescence after stimulation (ΔF/F_0_) and areas under the curve (AUCs) were calculated with Prism 9 software. AUC measurements were performed by integrating the area between F_0_ and F at all time points after stimulation up to a defined end point.

### 2.12. RNA-Seq

Total RNA was isolated from the cells using the Qiagen RNeasy Mini Kit (Qiagen 74104). For each sample, 700 ng of total RNA was then used in Illumina’s TruSeq Stranded mRNA Library kit (20020594). Libraries were sequenced on Illumina NextSeq 550 as paired-end 42 nt reads. Sequence reads were analyzed with the STAR alignment—DESeq2 software pipeline described in the Supplementary File S3.

### 2.13. Active Motif CUT&Tag

For the transcriptome study, samples were sent to Active Motif for CUT&Tag analysis. Initially, cells were incubated overnight with Concanavalin A beads along with 1 µL of the primary anti-H3K9Ac antibody per reaction (Active Motif, catalog number 39917). Following the secondary anti-rabbit antibody incubation (1:100), tagmentation was carried out at 37 °C using protein-A-Tn5. To terminate the tagmentation process, a mixture of EDTA, SDS, and proteinase K was added, and the reaction was incubated at 55 °C. Subsequently, DNA extraction and ethanol purification were performed followed by PCR amplification and barcoding using the Active Motif CUT&Tag kit (53160), facilitating the identification and differentiation of the DNA fragments. SPRI bead cleanup (Beckman Coulter) was then performed, and, finally, the resulting DNA libraries were quantified and subjected to sequencing on the Illumina NextSeq 550 (generating 8 million, 38 paired end).

For data analysis, reads were aligned using the BWA algorithm (mem mode; default settings) [30]. Duplicate reads were eliminated, and only reads that uniquely mapped with a mapping quality of greater than or equal to 1 and formed matched pairs were retained for further analysis. The alignments were extended in silico at their 3’ ends, resulting in fragments of 200 base pairs, which were then assigned to 32 nt bins across the genome. The resulting histograms, representing the genomic “signal maps,” were stored in bigWig files. To identify peaks, the MACS 2.1.0 algorithm was employed with a *p*-value cutoff of 10^−7^, without the use of a control file and with the inclusion of the “-nomodel” option. Peaks that were present in the ENCODE blacklist, known to be false ChIP-Seq peaks, were excluded from the analysis. The signal maps and peak locations served as input data for Active Motifs’ proprietary analysis program, which generated Excel tables containing comprehensive information on sample comparisons, peak metrics, peak locations, and gene annotations.

For differential analysis, read counts within all merged peak regions were determined using Subread. The replicates for each experimental condition were then compared using DESeq2 [31].

Other key software used: bcl2fastq2 (v2.20) (processing of Illumina base-call data and demultiplexing), Samtools (v0.1.19) (processing of BAM files), BEDtools (v2.25.0) (processing of BED files), wigToBigWig (v4) (generation of bigWIG files), Subread (v1.5.2) (counting of reads in BAM files for DESeq2).

### 2.14. Treatment of Cells

#### 2.14.1. S1P and S1PR_2,4_ Agonist Treatment

To confirm the role of S1P signaling, the results obtained in SGPL1-deficient astrocytes were recapitulated by extracellular administration of S1P (10 nM) to control astrocytes for 24 h. S1P stock solution was prepared in water.

To confirm the role of S1PR_2_ and S1PR_4_ for the activation of P2Y1R signaling, control astrocytes were treated with 5 µM of the specific agonist of S1PR_2_ (CYM5520) and of S1PR_4_ (CYM50308) for 24 h. CYM5520 and CYM50308 were both dissolved in DMSO, and the corresponding amount of DMSO was added to the untreated astrocyte cultures.

#### 2.14.2. P2Y1R Antagonist Treatment

The rescue experiments of astrocytic hyperactivity were conducted by treating astrocytes with 100 µM of the specific P2Y1R antagonist MRS2179 for 24 h. MRS2179 stock solution was prepared in water.

#### 2.14.3. P2Y1R Agonist Treatment

To confirm the role of P2Y1R in mediating astrogliosis, control astrocytes were treated with 5 nM of MRS2905, a specific P2Y1R agonist for 24 h.

### 2.15. Statistical Analysis

GraphPad Prism 9 was used for statistical analysis. Mean ± SEM values were presented in the figures, which were obtained from at least 3 independent experiments unless otherwise specified. In total, 3 independent experiments corresponded to different astrocyte isolations from pups of different breeding cages. Each independent experiment was run in triplicate, corresponding to astrocytes from 3 different pups. Student’s *t*-test with false discovery rate (FDR) correction or One-Way ANOVA with Bonferroni multiple comparison test was used to determine the significance of differences between experimental groups and controls, as appropriate. A *p*-value of less than 0.05 was considered statistically significant (* *p* < 0.05, ** *p* < 0.001, *** *p* < 0.0001, **** *p* < 0.0001, compared to the respective control group).

## 3. Results

### 3.1. Neural Ablation of SGPL1 Triggers Astrogliosis in Murine Brains via the P2Y1R

**Figure 1:**
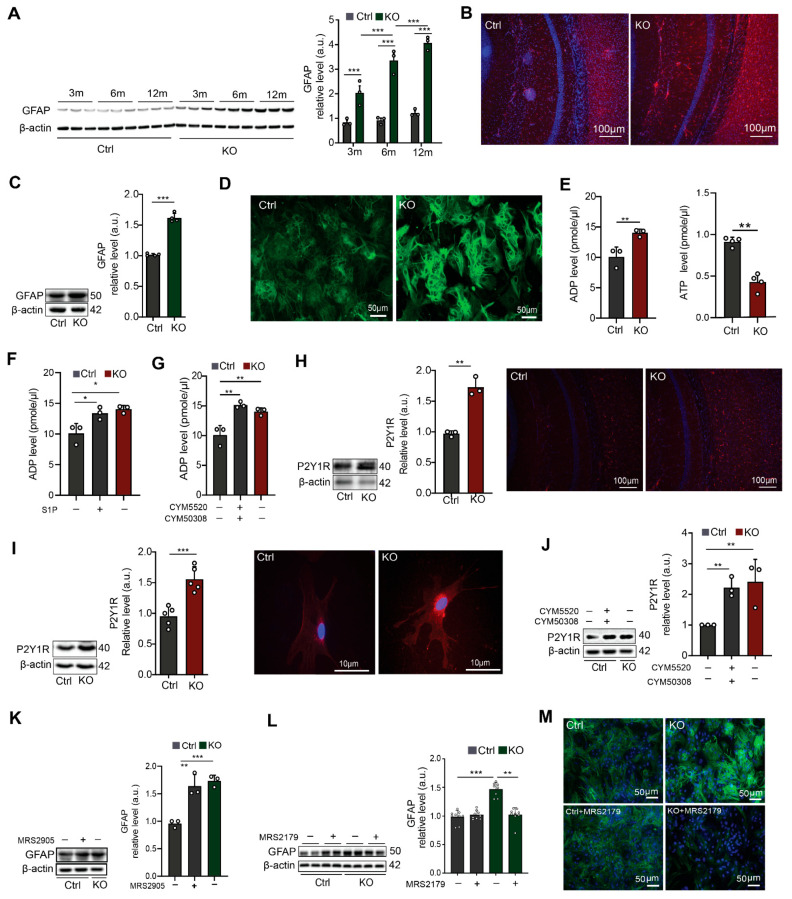
Neural ablation of SGPL1 triggers astrogliosis in murine brains via P2Y1 receptors. (**A**) Protein quantification of GFAP in cortices of control (Ctrl) and SGPL1-deficient (KO) mice of the indicated age in months (m). (**B**,**D**) Representative images of cortical slices and cultured astrocytes stained for GFAP from Ctrl and KO mice. (**C**) Protein quantification of GFAP in primary cultured astrocytes from Ctrl and KO mice. (**E**) Quantification of ADP and ATP in the culture media of astrocytes derived from Ctrl and KO mice. (**F**,**G**) Quantification of ADP level in the presence (+) and absence (−) of S1P (10 nM, 24 h) and of S1PR_2/4_ agonist (CYM5520 and CYM50308, 5 µM each) as indicated in the culture media of astrocytes derived from Ctrl and KO mice. (**H**,**I**) Protein quantification of P2Y1 receptor (P2Y1R) and representative images from cortical slices and cultured single astrocytes stained for P2Y1R from Ctrl and KO mice. (**J**) Protein quantification of P2Y1R in primary cultured astrocytes in the presence (+) and absence (−) of S1PR_2/4_ agonist (CYM5520 and CYM50308, 5 µM each) for 24 h as indicated. (**K**) Protein quantification of GFAP in primary cultured astrocytes in the presence (+) and absence (−) of P2Y1 agonist, (MRS2905, 5 nM) from Ctrl and KO mice. (**L**) Protein quantification of GFAP in the presence (+) and absence (−) of P2Y1 antagonist (MRS2179, 100 µM), as indicated from Ctrl and KO astrocytes. (**M**) Representative images of primary cultured astrocytes stained for GFAP from Ctrl and KO mice in presence (+) and absence (−) of P2Y1R antagonist (MRS2179, 100 µM). For all, representative immunoblots are shown with β-actin as loading control. Bars represent means ± SEM, (*n* ≥ 3; one way ANOVA and unpaired Student *t* test; * *p* < 0.05 ** *p* < 0.001, *** *p* < 0.0001).

To clarify if and how astrocytes were affected in SGPL1-deficient brains, we first monitored the expression of GFAP in the cortex of control and SGPL1^fl/fl/Nes^ mice at different ages. The considerable age-dependent increase in GFAP in brains lacking neural SGPL1 when compared to controls (Figure 1A) was the first indication that SGPL1 deficiency triggered astrogliosis. This result was then confirmed in primary cultured astrocytes, in which the contents of GFAP in SGPL1-depleted cells exceeded those of controls by more than 50% (Figure 1C). Moreover, immunostaining of cortical slices from 12-month-old mice and cultured astrocytes supported these findings (Figure 1B,D).

A plethora of data related to the involvement of purinergic mechanisms in astrogliosis induced by CNS injury [32]. We have recently shown an elevated production of ATP in SGPL1-deficient astrocytes [24]. We now measured the amount of extracellular ATP and ADP in control and SGPL1-deficient astrocytes. As shown in Figure 1E, the amount of ADP significantly increased by about 50% in astrocytes derived from SGPL1^fl/fl/Nes^ mice, whereas the amount of ATP was reduced accordingly (Figure 1E). To verify whether the S1P/S1PR_2,4_ signaling shown before to mediate the increased ATP production [24] is also responsible for the increased amount of extracellular ADP in SGPL1-deficient astrocytes, we treated control astrocytes for 24 h either with S1P (10 nM) or with 5 µM of specific agonists of S1PR_2_ and S1PR_4_, respectively. As shown in Figure 1F,G, administration of either S1P or of S1PR_2,4_ agonists were sufficient to reproduce the result obtained in SGPL1-depleted cells (Figure 1F,G), confirming that S1P signaling via its receptors S1PR_2_ and S1PR_4_ mediated the increased extracellular ADP level in SGPL1-deficient astrocytes. As ADP is primarily a ligand for the metabotropic purinergic P2Y1R, known to be highly expressed by reactive astrocytes [33], we first examined whether its expression was affected in SGPL1-deficient astrocytes. As illustrated in Figure 1H,I, the expression of P2Y1R increased by about 50% in the cortex of SGPL1-deficient mice, as well as in the cortical astrocytes. In addition, immunostaining of cortical slices of 12-month-old mice and of cultured astrocytes supported these findings (Figure 1H,I). The connection of S1P signaling through S1PR_2,4_ and the increased expression of P2Y1R was approved by incubating control astrocytes with specific agonists of S1PR_2,4_ (5 µM each). This treatment of controls yielded a result similar to that obtained in the SGPL1-depleted cells (Figure 1J).

To further confirm the functional connection between astrogliosis and the purinergic mechanism mediated by P2Y1R in SGPL1-deficient astrocytes, we determined GFAP expression in control astrocytes exposed for 24 h to MRS2905, a specific P2Y1R agonist (5 nM). As depicted in Figure 1K, a considerable, nearly identical increase in GFAP expression was detected in both treated control astrocytes and SGPL1-deficient astrocytes (Figure 1K). In addition, we treated the control and SGPL1-deficient astrocytes with MRS2179 (100 µM, 24 h), a selective P2Y1R antagonist, and examined the expression of GFAP by immunoblotting and immunostaining. As shown in Figure 1L,M, GFAP overexpression was almost completely ablated in treated SGPL1-deficient astrocytes (Figure 1L,M). Together these results indicate that the upregulation of GFAP expression in SGPL1-deficient astrocytes is mediated via P2Y1R signaling.

### 3.2. SGPL1-Deficient Astrocytes Exhibit Decreased Basal [Ca^2+^]_i_ and Elevated Calbindin Expression Mediated by P2Y1R

**Figure 2:**
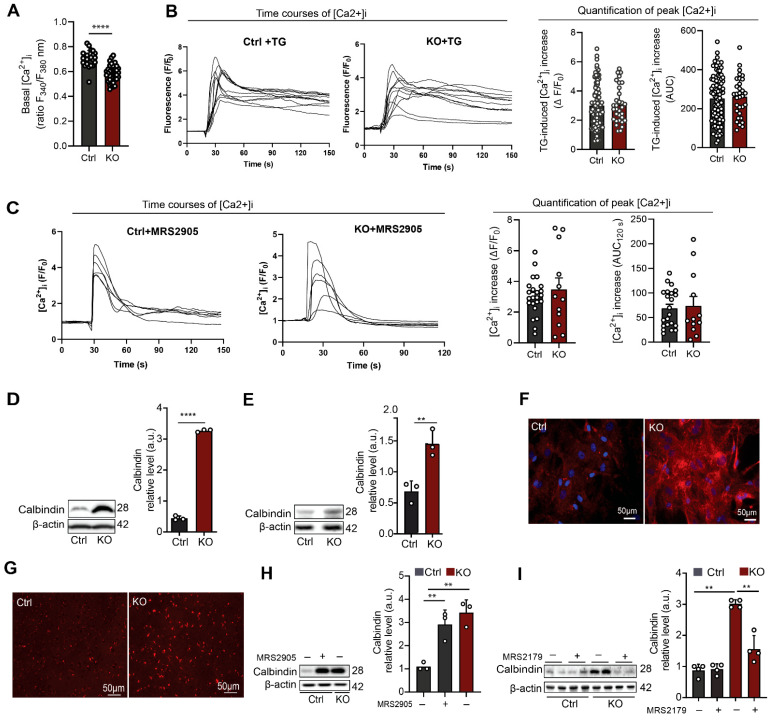
P2Y1R activation modulates calbindin expression in SGPL1-deficient astrocytes. (**A**) Basal [Ca^2+^]_i_ was measured in cells loaded with fura-2. Shown is the ratio of fluorescence emission at 340 and 380 nm of excitation. Each dot represents a single cell (Ctrl, control; KO, SGPL1-deficient). (**B**) Thapsigargin-induced [Ca^2+^]_i_ increases were measured in fluo-4-loaded cells stimulated with 1 μM thapsigargin (TG). Left: Representative time courses of [Ca^2+^]_i_ in individual Ctrl and KO cells. Fluo-4 fluorescence was normalized to baseline values (F/F_0_). Right: Quantification of peak [Ca^2+^]_i_ increases (ΔF/F_0_) and areas under the curve (AUCs; measured for 120 s after addition of thapsigargin). Each dot represents a single cell. (**C**) P2Y1R agonist-induced [Ca^2+^]_i_ increases were measured in fluo-4-loaded cells stimulated with 5 nM MRS2905. Left: Representative time courses of [Ca^2+^]_i_ in individual Ctrl and KO cells. Fluo-4 fluorescence was normalized to baseline values (F/F_0_). Right: Quantification of [Ca^2+^]_i_ induced by the P2Y1R agonist. Shown are peak [Ca^2+^]_i_ increases (ΔF/F_0_) and areas under the curve (AUCs; measured for 120 s after addition of agonist). Each dot represents a single cell. (**D**,**E**) Protein quantification of calbindin in primary cultured astrocytes and cortices of Ctrl and KO mice. (**F**,**G**) Representative images of astrocytes and cortical slices stained for calbindin from Ctrl and KO mice. (**H**) Protein quantification of calbindin in primary cultured astrocytes for 24 h in the presence (+) and absence (−) of P2Y1 agonist (MRS2905, 5µM) as indicated. (**I**) Protein quantification of calbindin in primary cultured astrocytes treated for 24 h in the presence (+) and absence (−) of P2Y1R antagonist (MRS2179, 100 µM) as indicated. For all, representative immunoblots are shown with β-actin as loading control. Bars represent means ± SEM, (*n* ≥ 3; one way ANOVA and unpaired Student *t* test; ** *p* < 0.001, **** *p* < 0.00001).

Astrogliosis is a multifaceted and intricate process that encompasses a range of molecular and morphological alterations in astrocytes, extending beyond a mere upregulation of GFAP expression [25,26,34]. Astroglial calcium signaling originating from endoplasmic reticulum is a key factor in initiating an astrogliotic response [35]. As purinergic receptors are key players in the mobilization of astrocytic calcium [36], we first measured the basal calcium level in control and SGPL1-deficient astrocytes. Surprisingly, basal calcium level was significantly decreased in SGPL1-deficient astrocytes (Figure 2A). Furthermore, thapsigargin-induced [Ca^2+^]_i_ increases were similar in SGPL1-deficient and control astrocytes, regarding both the maximum [Ca^2+^]_i_ increase and the AUC for 120 s (Figure 2B). Thus, endoplasmic reticulum calcium storage was not altered. Consistently, P2Y1R agonist administration evoked equal responses in control and SGPL1-deficient astrocytes (Figure 2C). Further examination revealed a considerable increase in calbindin both in SGPL1-deficient astrocytes as well as in cortices from 12-month-old SGPL1^fl/fl/Nes^ mice (Figure 2D,E). Immunostaining of cultured astrocytes (Figure 2F) and of cortical slices of 12-month-old mice (Figure 2G) confirmed these results. Calbindin is a member of the superfamily of calcium-binding proteins that was found to be highly expressed in reactive astrocytes [37]. Importantly, the results obtained in astrocytes lacking SGPL1 were replicated by incubating control astrocytes with the P2Y1R agonist, MRS2905 (5 nM) for 24 h (Figure 2H). Consistently, the administration of MRS2179, a selective P2Y1R antagonist (100 µM, 24 h), brought calbindin expression back to control levels (Figure 2I). These results suggested that the increased expression of calbindin was keeping cytosolic calcium levels in check in the SGPL1-deficient astrocytes [38].

### 3.3. In SGPL1-Deficient Astrocytes Transcriptional and Epigenetic Alterations Lead to Activation of the NLRP3 Inflammasome via a P2Y1R-Mediated Mechanism

**Figure 3:**
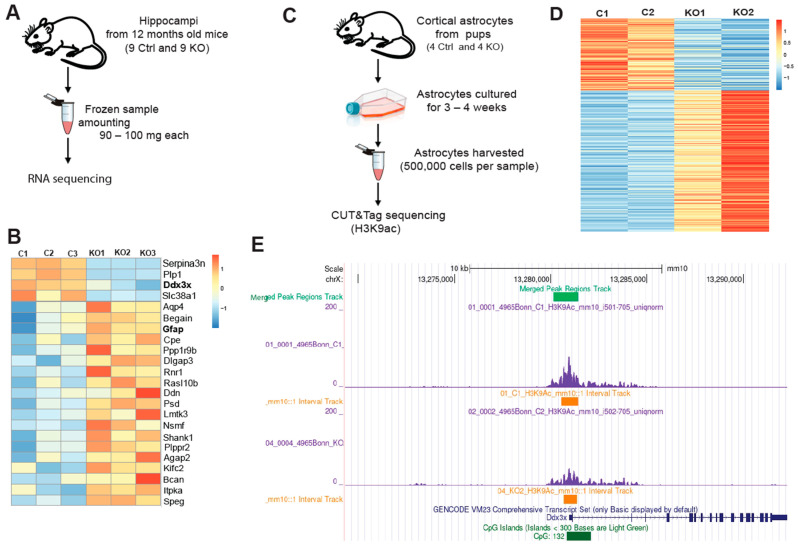
Transcriptome analysis in hippocampi and CUT&Tag chromatin profiling for histone H3K9ac in astrocytes. (**A**) Overview of RNA-Sequencing. (**B**) Heat map of differentially transcribed genes (see also Supplementary File S1). (**C**) Summary of CUT&Tag sequencing. (**D**) Heat map showing the intensity of H3K9ac signals across the astrocytic genome. Each sample shown contains astrocytes pooled from two individuals. (**E**) Visualization of a 10 kb chromatin segment of the Ddx3x promoter region showing the H3K9ac occupancy, using Integrated Genome Browser (see also Supplementary File S2).

Activation of astrocytes is often accompanied by intensified inflammatory pathways [34,39]. Based on the elevated histone acetylation in SGPL1-deficient astrocytes [22], and on the close interconnection between epigenetic regulation and inflammation [40], we first investigated gene transcription in brain tissue from SGPL1^fl/fl/Nes^ mice. Histone acetylation is an epigenetic hallmark of an open and accessible chromatin environment, usually permissive for and promoting active transcription [41]. The formerly observed increase in astrocytic H3K9 acetylation, a classical “activating” histone modification found at promoters, was indicative of changes in H3K9ac occupancy on promoter regions within the astrocytic genome, which may, in turn, affect transcription of the respective genes. To examine transcriptional state of genes affected by SGPL1 deficiency as well as their epigenetic statuses, we performed RNA sequencing (Figure 3A,B) together with CUT&Tag sequencing [42] for H3K9ac (Figure 3C–E) in hippocampi and cortical astrocytes. Figure 3B depicts differential transcription of selected genes out of 34 genes that were found to be affected (see Supplementary File S1). Among these genes, *Ddx3x* caught our particular attention, as its transcription as well as H3K9ac occupancy at the Ddx3x promoter differed notably between control and SGPL1-deficient samples in RNA-Seq and CUT&Tag (Figure 3E and Supplementary File S2).

**Figure 4:**
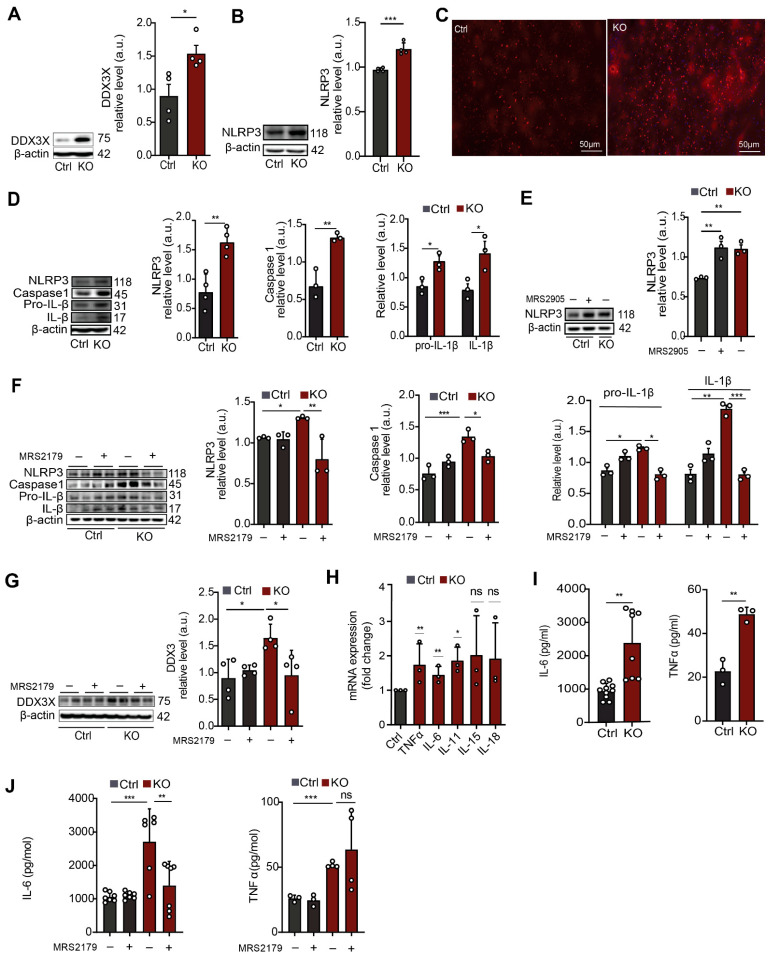
Neural SGPL1 ablation activates inflammation via P2Y1R in primary cultured astrocytes. (**A**,**B**) Protein quantification of DDX3X and NLPR3 in cortices of control (Ctrl) and SGPL1-deficient (KO) mice. (**C**) Representative images of cortical slices stained for NLPR3 from Ctrl and KO mice. (**D**) Protein quantification of NLRP3, Caspase1, Pro-IL-β, and IL-β in primary cultured astrocytes of Ctrl and KO mice. (**E**) Protein quantification of NLRP3 in primary cultured astrocytes for 24 h in the presence (+) and absence (−) of P2Y1R agonist (MRS2905, 5 nM) as indicated. (**F**,**G**) Protein quantification of NLRP3, Caspase1, Pre-IL-β and IL-β, and DDX3X in the primary cultured astrocytes treated for 24 h in the presence (+) and absence (−) of P2Y1R antagonist (MRS2179, 100 µM) as indicated. (**H**) Relative mRNA transcripts of the indicated cytokines in SGPL1-deficient astrocytes assessed by qPCR. (**I**,**J**) Expression of IL-6 and TNFα in Ctrl and KO astrocytes with (+) and without (−) P2Y1 antagonist (MRS2179, 100 µM) treatment. For all, representative immunoblots are shown with β-actin as loading control. Bars represent means ± SEM, (*n* ≥ 3; one way ANOVA and unpaired Student *t* test; * *p* < 0.01, ** *p* < 0.001, *** *p* < 0.0001).

Although CUT&Tag sequencing revealed slightly decreased H3K9ac, and RNA-Seq suggests a lower amount of Ddx3x mRNA in SGPL1-deficient astrocytes, immunoblot experiments showed a significant increase in DDX3X protein level (Figure 4A). These results suggested a possible feedback loop between DDX3X protein level and transcriptional regulation, in which high DDX3X may have led to a downregulation of transcription of the respective gene. While our epi-genomic and transcriptomic findings at the Ddx3x locus did not match with our observation of elevated protein levels of DDX3X, it was evident that the absence of SGPL1 had a significant impact on gene regulation and transcription, as well as on protein levels. Note that the gene encoding the protein DDX3X was shown to activate NLRP3 inflammasome [43]. Consistent with the elevated amounts of DDX3X, the expression of the NLRP3 inflammasome was significantly increased in cortices from 12-month-old SGPL1^fl/fl/Nes^ mice (Figure 4B,C), as well as in astrocytes generated thereafter (Figure 4D). As shown in Figure 4D also the expression of caspase 1, the effector subunit of the NLRP3 inflammasome required for the processing and secretion of the inflammatory cytokine IL-1ß, was increased in SGPL1-depleted astrocytes (Figure 4D). Similarly, the expression of pro-IL-1ß and IL-1ß was elevated in these astrocytes (Figure 4D).

Administration of the specific P2Y1R agonist, MRS2905 (5 nM, 24 h) increased NLRP3 expression in control astrocytes, recapitulating the effect observed in SGPL1-depleted cells (Figure 4E). Consistently, treatment of astrocytes with MRS2179, a P2Y1R antagonist (100 µM, 24 h), reversed the expression of NLRP3, caspase-1, and IL-1β of the SGPL1-deficient astrocytes (Figure 4F). The expression of DDX3X was also reversed in the presence of the P2Y1R antagonist (Figure 4G). These findings document that the NLRP3 inflammasome activation in SGPL1-deficient astrocytes are mediated by P2Y1R signaling. Finally, we investigated the mRNA expression of inflammatory cytokines, including IL-6, IL-11, IL-15, IL-18, and TNFα, known to promote astrogliosis and the formation of glial scar [26]. We indeed found increased transcripts of IL-6, IL-11, and TNFα in primary cultured astrocytes lacking SGPL1 (Figure 4H). Moreover, we detected increased secretion of IL-6 and TNF in the culture medium of SGPL1-deficient astrocytes (Figure 4I). Treatment with the P2Y1R antagonist MRS2179 (100 µM, 24 h) rescued only IL6 but not TNFα secretion (Figure 4J).

Together, our findings convincingly documented that P2Y1R is a key player in astrogliosis and NLRP3 activation in SGPL1-deficient brains.

## 4. Discussion

The focus of the present study was to unravel the consequences of SGPL1 deficiency in astrocytes. Although astrocytes are considered as cells of high degrees of plasticity that may alter their properties in a culture medium [32], we considered it an appropriate model, as our previous results obtained in brain tissue could be recapitulated in primary cultured astrocytes [22]. Astrocytes are organized in functional networks that exert many complex essential functions in a healthy brain, including the support of synaptic function and plasticity, allocation of energy substrates, and regulation of regional cerebral blood flow [44]. Thus, any alteration in brain homeostasis triggers astrocyte reactivity, a process known as reactive astrogliosis, which constitutes a sensitive hallmark for several if not all brain pathologies, including ischemia, infections, stroke, and neurodegenerative disorders [26,34,39]. Given the affected neuronal health in SGPL1-deficient brains [20,21], detection of reactive astrogliosis, as confirmed by an age-dependent upregulation of GFAP expression, was not unexpected. Moreover, S1P/S1PR signaling axis has been shown before to control glial activation in mice with Sandhoff disease and was assumed to be generally important during the pathogenesis of neurodegenerative diseases [45].

The involvement of abnormal S1P metabolism in neuroinflammation along with neuronal damage and memory deficits is not new. It has also been shown in rats injected with lipopolysaccharides or beta-amyloids [46]. Also, S1P signaling specifically through S1PR_1_ was associated with astrogliosis and neuroinflammation [47,48]. In another experimental setting, simulated inflammation in primary cultured murine astrocytes detected S1PR_3_ to be responsible for the S1P-promoted inflammatory response [49]. Accordingly, astrocytic S1PR_1_ and S1PR_3_ were upregulated in multiple sclerosis lesions [50,51]. However, in SGPL1-deficient brains, S1P derived from astrocytes activates the microglia via S1PR_2_, thus triggering the release of inflammatory cytokines [23]. Here, we show that the S1P/S1PR_2/4_ signaling axis stimulates the purinoreceptor P2Y1R in SGPL1-deficient astrocytes, which mediates the activation of the NLRP3 inflammasome, a central player in neuroinflammation [52]. The NLRP3 inflammasome is a critical component of the innate immune system that mediates caspase-1 activation and the secretion of proinflammatory cytokines, including IL-1ß, in response to microbial infection and cellular damage [53]. Activation of the NLRP3 inflammasome usually comprises two signals, a priming and an activation signal [53]. The priming signal is triggered by several factors, including damage- and/or pathogen-associated molecular patterns (DAMPS/PAMPS) but also cytokines, resulting in increased expression of *Nlrp3*, *Caspase*-*1*, *pro*-*IL*-*1ß*, and *IL*-*1ß* [54]. In SGPL1-deficient astrocytes, cytokines released by microglia [23] might be involved in priming the production of the different inflammasome components. However, our results obtained with the specific agonist and antagonist of P2Y1R argue in favor of a mechanism involving purine nucleotides [54]. Yet, ATP-like beta-amyloids (the main component of senile plaques in Alzheimer disease), as well as several other factors, are rather stimulating the activation signal that initiates the assembly and thus the active state of the inflammasome [54]. The latter triggers caspase-1 to cleave pro-IL-1ß, yielding the release of IL-1ß [53]. Our results indicated that the stress protein DDX3X is involved in the activation of the NLRP3 inflammasome in SGPL1-deficient astrocytes, as recently shown in primary bone-marrow-derived macrophages [43]. Note that DDX3 is a multifunctional protein that participates in translational control of inflammation induced by infections and injuries [55]. Recently, the involvement of DDX3 in motor neuron degeneration in amyotrophic lateral sclerosis has been reported [56].

**Figure 5:**
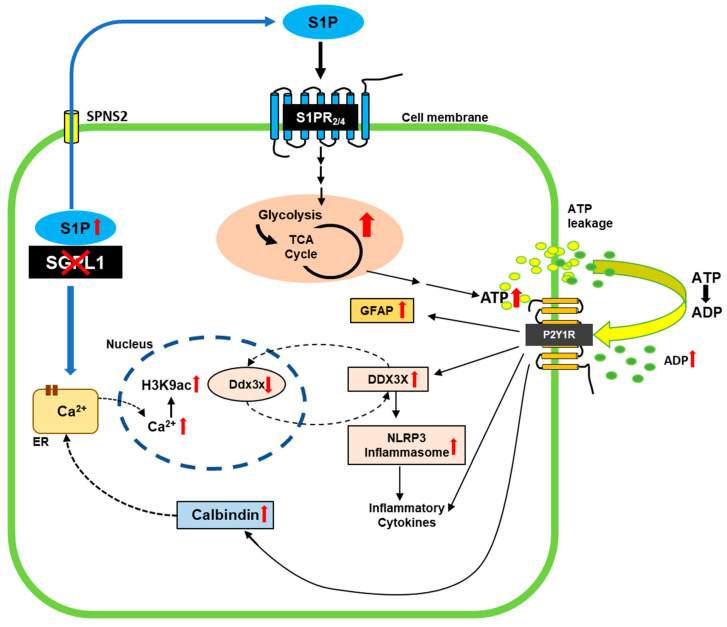
Scheme of the effects of SGPL1 ablation in astrocytes. In the absence of SGPL1, accumulated S1P is secreted by the cells [23]. By binding to S1PR_2,4_, it elicits signaling cascades that promote increased expression of proteins involved in glucose breakdown via glycolysis and the TCA [24], finally leading to an increased amount of extracellular ADP, a ligand of P2Y1R. Shown are the implications of this receptor in the gliotic and hence proinflammatory response of SGPL1-deficient astrocytes. In addition, P2Y1R signaling triggers the expression of calbindin, which binds cytosolic Ca^2+^ released from the endoplasmic reticulum as a result of S1P accumulation [67]. Finally, nuclear Ca^2+^ promotes H3K9 acetylation [22] that affects the transcription of Ddx3x. The increased expression of DDX3X suggests a possible feedback loop between protein level and transcriptional regulation. See text for further explanations.

Based on the critical role of calcium mobilization in the activation of the NLRP3 inflammasome [57], we reasoned that the S1P-induced release of calcium from the endoplasmic reticulum [20,22] might trigger the inflammatory responses of astrocytes, as shown in SGPL1-dficient neurons. Yet, astrocytes in contrast to neurons evidently develop a coping strategy that triggers the production of the cytosolic calcium buffer calbindin. However, calbindin was also shown to act as a calcium signal modulator [38]. Based on its fast binding sites, calbindin is able to affect the early rising phase of Ca^2+^ transients [38], known to be evoked by P2Y1R signaling in astrocytes [58]. Intriguingly, our data indicate that P2Y1R signaling mediates the elevated calbindin expression in SGPL1-deficient astrocytes (Figure 5), suggesting that it has a role to play beyond calcium binding. Although we cannot conclusively explain at present time, in the activation of NLRP3 inflammasome, we observed increased releases not only of IL-1ß but also of other proinflammatory cytokines, including IL-6, IL-11, and TNFα. Of interest, the NLRP3 inflammasome was shown to be involved in the pathoautoimmune GFAP astrocytopathy and was proposed as a marker for the severity of the disease and as a potential therapeutical target [59]. The data of the present study identified the metabotropic purinoreceptor P2Y1R in directly regulating both GFAP expression and NLRP3 activation in SGPL1-deficient astrocytes (Figure 5). The key to the activation of P2Y1R is the increased amount of extracellular ADP, which is a result of the S1P/S1PR_2/4_ signaling axis in the SGPL1-deficient astrocytes. We have recently shown that predominantly glycolytic astrocytes [60] in the absence of SGPL1 become aerobic, owing to S1P/S1PR_2/4_ signaling, producing an excess of ATP [24]. The increase in extracellular ADP, as shown here, is most probably a result of the ATP released into the extracellular medium, where it is degraded by ectonucleotidases, yielding ADP. Purine-induced reactive astrogliosis is well-known [61]. Extracellular nucleotides acting through the seven ionotropic P2X and the eight G protein-coupled P2Y receptors expressed on astroglia and microglia were also reported to mediate a gliotic response [62]. Among these receptors, astrocytic P2 receptors were assumed to mediate reactive astrogliosis, a reaction contributing to neuronal death in neurodegenerative diseases [62]. Accordingly, astrogliosis was often induced by treating astrocytes with ATP or its structural analogs [61,63,64,65]. Moreover, a micro-infusion of 2-methylthio ATP (2-MeSATP) into the rat nucleus accumbens resulted in elevated expressions of GFAP, astrogliosis, and CNS injury, whereas treatment with its antagonists, reactive blue 2 and pyridoxal-phosphate-6-azophenyl-2,4-disulphonic acid (PPADS), counteracted 2-MeSATP-induced astrogliosis, supporting the hypothesis that purine nucleotides are involved in these processes via the stimulation of P2 receptors in vivo [64]. Likewise, the ATP-induced astrogliosis was counteracted by PPADS in cultured astrocytes [65]. Consistently, treatment of SGPL1-deficient astrocytes with MRS2179, a known P2Y1R antagonist, reversed the expression of GFAP to control levels. A recent study performed in an Alzheimer’s disease mouse model emphasized that astroglial network dysfunction is mediated by purinergic signaling via P2Y1R [33]. Moreover, this study shows that P2Y1Rs are strongly expressed by reactive astrocytes surrounding the senile plaques and excludes the role of P2X receptors for astrocytic hyperactivity [33]. According to augmented cortical ADP concentrations reported to be essential for purinergic signaling via P2Y1R in the Alzheimer’s disease mouse model [33], we show here significantly increased ADP levels in the medium of SGPL1-deficient astrocytes. It is well established that different cell types in the brain release ATP, known to function as a neurotransmitter, neuromodulator, in astrocyte-to-neuron communication, propagating astrocytic responses, and formatting microglia responses [66]. Upon catabolism of extracellular ATP by ectonucleotidases, other adenosine receptors, including the ADP-activated P2Y1R, contribute to the described astrocytic and microglia responses [66]. Sustained, abnormal elevated levels of extracellular ATP, and hence of ADP and adenosine signaling via P2X7R, P2Y1R and A_2A_R, respectively, are closely associated with brain disorders and are particularly involved in brain damage and dysfunction [33,66]. However, neither the source of extracellular ATP nor the mechanism of its release upon noxious brain conditions has yet been clarified [66]. In primary cultured astrocytes derived from SGPL1^fl/fl/Nes^ mice, the increased amount of extracellular ADP depends on S1P signaling via its receptors, S1PR_2,4_ [24].

## 5. Conclusions

In this study, we found that astrocytes undergo reactive astrogliosis along with activation of the NLRP3 inflammasome in SGPL1-deficient brains. On a molecular level, ADP-induced activation of P2Y1R signaling turned out to be central to these processes. We show that P2Y1R is directly involved in the expression of the stress protein DDX3X, which contributes to the activation of the NLRP3 inflammasome (Figure 5). The latter triggers caspase-1 activity and thus the generation of additional proinflammatory cytokines. Furthermore, P2Y1R signaling appears to be involved in the coping strategy that allows astrocytes to keep cytosolic Ca^2+^ levels in check.

To the best of our knowledge, this is the first study that shows how changes in S1P metabolism and hence signaling contribute to neuroinflammation induced by the activation of the NLPR3 inflammasome in astrocytes. Our data may therefore initiate new therapeutic strategies surrounding S1P metabolism and signaling. Note that no therapeutic drugs targeting astrocytic S1PR2/4 and/or P2Y1R are in clinical use so far. However, the P2Y1R antagonist MRS2179 has been proposed as a lead structure for antithrombotic drugs due to its antiaggregatory activity [68], whereas P2Y1R agonists emerged as attractive therapeutic drug candidates for the treatment of diabetes type 2 [69].

## Data Availability

All data generated or analyzed during this study are included in this published article.

## Supplementary Materials

The following supporting information can be downloaded at: https://www.mdpi.com/article/10.3390/cells12141844/s1; Supplementary File S1: Excel sheet showing the RNA seq results; Supplementary File S2: Excel sheet showing the results of CUT&Tag; Supplementary File S3: Explanation of Data—RNA Sequencing Analysis.
