# Supplementary material for: S1P Lyase Deficiency in the Brain Promotes Astrogliosis and NLRP3 Inflammasome Activation via Purinergic Signaling
Source: Cells. 2023 Jul 13;12(14):1844. doi: 10.3390/cells12141844 (PMC10378183; doi:10.3390/cells12141844)
Supplement: Supplementary file 1 [file cells-12-01844-s001.zip › cells-2404361-Supplementary File S3.pdf]

Explanation of Data – RNA Sequencing Analysis

I. Data Delivery

Data and analysis files are posted to a customer FTP account, which can be accessed with the provided username and password. We recommend using the Filezilla client software to download the data (https://filezilla-project.org/). When using Filezilla, FTP data transfer will be encrypted by the TLS connection. Please download the files promptly and store in a safe place, preferable with backup. Raw read data (FASTQ files) and possibly other processed data files will be required for submission to the NCBI/GEO data repository.

NOTE: When using Filezilla Quickconnect, the Port field can be left empty:

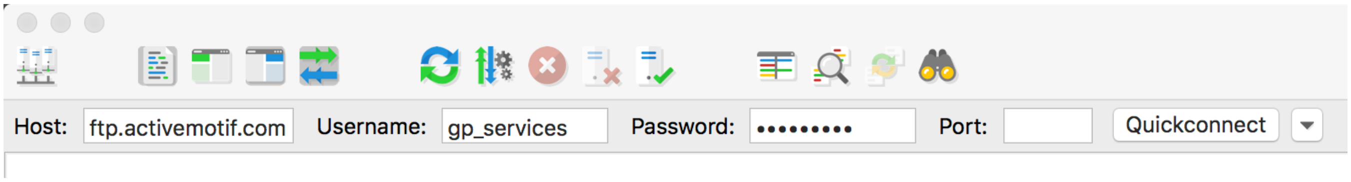

II. Data Files

The following data files are posted to the customer FTP account:

1. FASTQ. Files with extension *.fastq.gz. Compressed files containing Illumina chastity-filtered reads with phred+33 quality scores. Each sample has 2 files: read1 (_r1) and read2 (_r2).
2. BAM. Files with extension *.bam. Binary files containing reads and alignment information. For more information on the generic BAM format see http://samtools.sourceforge.net/.
3. BAM-index. Small files with extension *.bam.bai. These are index files that need to be located in the same directory as the BAM file for certain BAM file visualizations.

Other formats or intermediate files can be provided upon request. Please inquire.

III. Analysis Files

The following files and subfolders are generated by Active Motif's Analysis Program and are posted in a zip-compressed folder (typically named "AM Institution RNA-Seq Quote#"):

1. EXCEL TABLES – aggregate tables compiling all the key metrics for all samples and differential results of all comparisons for this RNA-Seq project.
2. DIFF_ANALYSIS – differential analysis results using the DESeq2 software package, containing in separate subfolders the results for each of the pairwise comparisons, including:
    a. DESeq2 output tables (csv) and summary and stats files

Active Motif, Inc. • 1914 Palomar Oaks Way, Suite 150 • Carlsbad, CA 92008 • *toll free* 877.222.9543 • *fax* 760.431.1351
www.activemotif.com

   b. GRAPHICS -- set of standard graphics showing differential results (described in a separate document)
   c. GSEA -- gene set enrichment analysis results using the GSEA software package
3. GRAPHICS – set of standard graphics showing data for all samples (see separate document).
4. SUPPLEMENTARY FILES – a collection of additional output files.

Detailed descriptions of all files can be found in Section V.

IV. Description of Analysis Steps

1. Read Mapping: The paired-end 42 bp sequencing reads (PE42) generated by Illumina sequencing (using NextSeq 500) are mapped to the genome using the STAR algorithm with default settings. Alignment information for each read is stored in the BAM format.
2. Fragment Assignment: The number of fragments overlapping predefined genomic features of interest (e.g. genes) are counted. Only read pairs that have both ends aligned are counted. Read pairs that have their two ends mapping to different chromosomes or mapping to same chromosome but on different strands are discarded. The gene annotations we use were obtained from Subread package. These annotations were originally from NCBI RefSeq database and then adapted by merging overlapping exons from the same gene to form a set of disjoint exons for each gene. Genes with the same Entrez gene identifiers were also merged into one gene.
3. Differential Analysis: After obtaining the gene table containing the fragment counts of genes, we perform differential analyses to identify statistically significant differential genes using DESeq2. The following lists the pre-processing steps before differential calling.
   a. Data Normalization: DESeq2 expects un-normalized count matrix of sequencing fragments. The DESeq2 model internally corrects for library size using their median-of-ratios method. The gene table obtained from Analysis Step 2) is used as input to perform the DESeq2's differential test.
   b. Filtering before multiple testing adjustment: After a differential test has been applied to each gene except the ones with zero counts, the p-value of each gene is calculated and adjusted to control the number of false positives among all discoveries at a proper level. This procedure is known as multiple testing adjustment. During this process, DESeq2 by default filters out statistical tests (i.e. genes) that have low counts by a statistical technique called independent filtering. It uses the average counts of each gene (i.e. baseMean), across all samples, as its filter criterion, and it omits all genes with average normalized counts below a filtering threshold from multiple testing adjustment. This filtering threshold is automatically determined to maximize detection power (i.e. maximize the number of differential genes detected) at a specified false discovery rate (FDR). In the spreadsheet listing the summary of statistics (DESeq2_stats.xlsx), one can refer to "Genes with low counts" to get an idea of how many genes were omitted in the

Active Motif, Inc. • 1914 Palomar Oaks Way, Suite 150 • Carlsbad, CA 92008 • *toll free* 877.222.9543 • *fax* 760.431.1351
www.activemotif.com

process of multiple testing adjustment. The spreadsheet "DESeq2_results_allgene.csv", lists all genes, including genes with weak signals have valid p-values but "NA" adjusted p-values.

For a project with no replicates: the statistical tests cannot be performed, and information on p-values and FDRs cannot be provided.

c. Differential Calling: In an experiment with replicates, differential genes are detected by DESeq2 at 0.1 (or 10%) FDR (i.e. adjusted p-value). In an experiment without replicates, differential genes are defined using shrunkenLog2FC cutoff off 0.3. This 0.3 cutoff is arbitrary, and it is best to interpret the results with prior biological knowledge.

4. Gene Set Enrichment Analysis (GSEA): Using DESeq2 normalized gene counts, we perform GSEA with default settings to determine whether members of *a priori* defined gene set based on biological knowledge (e.g. genes sharing the same GO category) are enriched. Before running GSEA, we add a small pseudo-count to the normalized counts to avoid divide by zero errors. Our standard GSEA analysis uses MSigDB's C5 (GO gene set) collection. Due to KEGG licensing terms, we no longer offer support for MSigDB's C2 (curated gene sets) collection. More information about MSigDB gene sets can be found at: https://www.gsea-msigdb.org/gsea/msigdb/.

V. Description of Analysis Files.

AT TOP LEVEL:

1. DESEQ2 RESULTS AGGREGATE TABLE ("00xyz_DESeq2_results_allgenes.xlsx"): An Excel file containing DESeq2-normalized gene counts and the results of the performed differential analyses.

Column Header	Information
Gene ID	NCBI Entrez Gene identification number
Gene Symbol	Short gene name (symbol)
Chr	Chromosome number(s) for all exons used for assigning gene counts
Start	Exon start position(s) for all exons used for assigning gene counts
End	Exon end position(s) for all exons used for assigning gene counts
Strand	Strand(s) for all exons used for assigning gene counts
Length	Total number of non-overlapping bases for all exons belonging to the same gene
Samples (Counts)	DESeq2-normalized counts for all samples
shrunkenLFC	DESeq2-shrunken log2 fold changes for each differential analysis
padj	Adjusted p-value for each differential analysis

2. DESEQ2 RESULTS FILTERED TABLES ("00xyz_DESeq2_results_*[comparison-cutoffs]*.xlsx"): Depending on the project, derived tables containing only differentially expressed genes can be provided. The filtering criteria used are indicated in the file name(s) (e.g. padj0.1, shrLFC1).

Active Motif, Inc. • 1914 Palomar Oaks Way, Suite 150 • Carlsbad, CA 92008 • *toll free* 877.222.9543 • *fax* 760.431.1351
www.activemotif.com

IN SUBDIRECTORY “DIFF_ANALYSIS”:

1. DESEQ2 RESULTS ALL (“DIFF_ANALYSIS/Comparison/ DESeq2_results_allgenes.csv”): A CSV file containing DESeq2-normalized gene counts and the results of a specific differential analysis. One file is provided per differential analysis.

Column Header	Information
Gene ID	NCBI Entrez Gene identification number
Gene Symbol	Short gene name (symbol)
Chr	Chromosome number(s) for all exons used for assigning gene counts
Start	Exon start position(s) for all exons used for assigning gene counts
End	Exon end position(s) for all exons used for assigning gene counts
Strand	Strand(s) for all exons used for assigning gene counts
Length	Total number of non-overlapping bases for all exons belonging to the same gene
Samples (Counts)	DESeq2-normalized counts for all samples
Group Mean (i.e. Control or Treatment)	Average of normalized counts for each sample group. The group name comes from the “metadata.csv” file.
baseMean	Average of normalized counts across all samples
rawLog2FC	Log2 fold changes before shrinkage by DESeq2. A pseudo-count of 1 is added to avoid divide by zero errors.
shunkenLog2FC	DESeq2-shrunken log2 fold changes for the differential analysis. The raw log fold change is shrunken to reduce exaggerated log fold changes from weakly expressed genes.
lfcSE	Standard error of the log2 fold change estimate. Only provided if replicates are available.
stat	Wald statistic, used to determine p-values by DESeq2. Only provided if replicates are available.
pvalue	Unadjusted p-value for the differential analysis. Only provided if replicates are available. “NA” values typically indicate that all counts for a gene are equal to zero, or the gene was excluded from the analysis due to an extreme count outlier.
padj	Adjusted p-value for the differential analysis. Uses the Benjamini-Hochberg FDR correction method. Only provided if replicates are available.

2. DESEQ2 RESULTS DIFF (“DIFF_ANALYSIS/Comparison/DESeq2_results_difgenes.csv”): A CSV file containing DESeq2-normalized gene counts and the results of a specific differential analysis. This file is a restricted version of the “allgenes.csv” file to highlight genes identified as differential using an adjusted p-value cutoff of 0.1 or a shrunken log fold change of 0.3. One file is provided per differential analysis. The columns contain the same information as described above for the “ALLGENES” table.

Active Motif, Inc. • 1914 Palomar Oaks Way, Suite 150 • Carlsbad, CA 92008 • *toll free* 877.222.9543 • *fax* 760.431.1351
www.activemotif.com

3. DESEQ2 SUMMARY ("DIFF_ANALYSIS/Comparison/DESeq2_summary.txt"): A text file summarizing the number of differential, outlier, and low count genes.

4. DESEQ2 STATS ("DIFF_ANALYSIS/Comparison/DESeq2_stats.xlsx"): An Excel file summarizing the number of up- and down-regulated differential genes. Also contains similar statistics as in the DESeq2 summary text file.

5. GSEA INPUT FILES ("DIFF_ANALYSIS/Comparison/gsea_input.[gct/cls]"): Text files that are the inputs for GSEA (https://www.gsea-msigdb.org/gsea/index.jsp). Each row in the gene cluster text file (.gct) contains the gene ID, gene symbol, and normalized expression levels on a per sample basis. In order to run the GSEA analysis, a small pseudo-count (0.00001) is added to the normalized expression levels. The categorical class file (.cls) defines the sample groupings present within the dataset. More information on these file formats can be found at:
   https://software.broadinstitute.org/cancer/software/gsea/wiki/index.php/Data_formats.

6. GSEA COMMAND ("DIFF_ANALYSIS/Comparison/gseacmd.txt"): The exact command line arguments used to run GSEA. When GSEA is installed locally, this command can be run to repeat the analysis.

7. GSEA RESULTS ("DIFF_ANALYSIS/Comparison/GSEA"): GSEA outputs. The GSEA analysis report (index.html) is the summary file and contains links to gene sets that are enriched within each experimental group. More details about output files can be found in GSEA's User Guide:
   https://www.gsea-msigdb.org/gsea/doc/GSEAUserGuideFrame.html).

IN SUBDIRECTORY "SUPPLEMENTAL FILES":

1. SEQ STATS ("Supplementary_Files/seq_stats.csv"): A CSV file summarizing alignment statistic and, if applicable, the number of differential genes identified for each differential analysis.

2. STAR REPORT ("Supplementary_Files/star_report.csv"): A CSV file summarizing detailed alignment statistics from the STAR aligner.

3. PROJECT STATS ("Supplementary_Files/Project Stats.csv"): A CSV file containing a more restrictive version of the STAR aligner report.

4. FEATURECOUNTS RESULTS ("Supplementary_Files/featureCounts.txt"): A text file containing the number of fragments assigned to individual genes. These counts are not normalized.

5. FEATURECOUNTS SUMMARY ("Supplementary_Files/featureCounts.txt.summary"): A text file containing summary statistics for featureCounts' assignment of fragments to genes.

Active Motif, Inc. • 1914 Palomar Oaks Way, Suite 150 • Carlsbad, CA 92008 • *toll free* 877.222.9543 • *fax* 760.431.1351
www.activemotif.com

6. FEATURECOUNTS LOG ("Supplementary_Files/out.fc.txt"): A text file containing a log of the featureCounts analysis.

7. FPKM MATRIX ("Supplementary_Files/gene_matrix_fpkm.txt"): A text file containing gene level counts, normalized using the FPKM (Fragments Per Kilobase of transcript per Million mapped reads) method.

8. METADATA ("Supplementary_Files/metadata.csv"): A CSV file specifying the group assignments used for the DESeq2-based differential analysis. The "all_groups" column is used for generating summary graphics, the "two_groups" columns are used to generate DESeq2-normalized counts, and the remaining columns are the assignments for each performed differential analysis.

NOTE: The graphics provided in the two or more "GRAPHICS" directories – one at the top level for graphics showing data for all samples, and one inside each differential analysis subfolder -- are described in a separate explanation document.

VI. Key Software Used

- STAR (v2.5.2b): https://github.com/alexdobin/STAR/blob/master/doc/STARmanual.pdf
- featureCounts in Subread software packages (v1.5.2) http://bioinf.wehi.edu.au/subread-package/SubreadUsersGuide.pdf
- DESeq2 (v1.14.1): https://bioc.ism.ac.jp/packages/2.14/bioc/vignettes/DESeq2/inst/doc/beginner.pdf
- GSEA (v4.0.3): http://software.broadinstitute.org/gsea/doc/GSEAUserGuideFrame.html

© 2020 Active Motif, Inc. All rights reserved.

Active Motif, Inc. • 1914 Palomar Oaks Way, Suite 150 • Carlsbad, CA 92008 • *toll free* 877.222.9543 • *fax* 760.431.1351
www.activemotif.com
